# Acknowledgment to Reviewers 2016

**DOI:** 10.1259/bjrcr.20179001

**Published:** 2017-02-14

The Editors gratefully acknowledge the contribution of the following colleagues who acted as reviewers in 2016.

Mohamed Abd Ellah

Shahram Abdi

Sharif Abdullah

Nasreddin Abolmaali

Mohamed Abou El-Ghar

Olu Adesanya

Himanshu Agarwal

Abhinav Aggarwal

Francesco Agnello

Anjali Agrawal

Fathinul Fikri Ahmad Saad

Zahoor Ahmed

Mohamed Nadeem Ahmed

Suhas Aithal Sitharama

Sami Akbulut

Sarah Alessi

Khalid Ali

Rosemary Allan

Robert Allison

Ali Alsafi

Michele Altiero

Matheus Alvarez

Roberta Ambrosini

Jayajothi Anandapadmanabhan

Mark Anderson

Renato Argirò

Ankur Arora

Abhishek Arora

Supreeta Arya

Julian Atchley

Swapndeep Atwal

Fred Avni

Shuting Bai

Alan Bainbridge

Ashank Bansal

Philip Bearcroft

Dietrich Beitzke

Jane Belfield

Daniel Bell

Davide Bellini

Venkatraman Bhat

Raj Bhat

Aashish Bhatt

Cusati Bianca

Margaret Bidmead

Justin Bishop

Ivana Blazic

Giorgio Bocchini

Antonia H.M. Bouts

Kate Broome

Anna Burch

Mohammad Butt

Stephen Butterfield

Alistair Calder

Luigi Camera

Gianpaolo Carrafiello

Albert Cendikiawan

Alfonso Cerase

Rosemary Chambers

Anthony Chambers

Anil Chauhan

Udit Chauhan

Ashish Chawla

Li Shyan Ch’ng

Anne-Marie Coady

Peter Corr

Renzo Corvò

Bruno Coulier

Luca Cozzi

Mary Crofton

John Curtis

Giancarlo d’Andrea

Çağrı Damar

Karthikeyan Damodharan

Edward Damrose

Felice D’arco

Tilak Das

James Davies

Andrew Degnan

Gianluca Deiana

Jun Deng

Lorenzo Derchi

Curtiland Deville

Louise Dickinson

Manjiri Dighe

Mainak Dutta

Mahmut Duymuş

David Eaton

David Elias

Amlyn Evans

Joanna Fairhurst

Beatrice Feragalli

Hilary Fewins

Bernie Foran

Colm Forde

Olivia Francies

Liana Franciscatto

Jurgen Futterer

Frank Gaillard

Shaileshkumar Garge

Francesco Gentili

Nitin P Ghonge

Matthew Gibson

Anthony Goode

Richard Graham

Roberto Grassi

Yvette Griffin

Jochen Grimm

Shabnam Grover

Mark Guelfguat

Susanna Guerrini

Franco Guida

Windsor Gunawardena

Louise Haine

Arsany Hakim

Thomas Hall

Sebastiaan Hammer

Athar Haroon

Christopher Harvey

John Harvey

Daichi Hayashi

Francine Hehn

Melanie Hiorns

Takashi Hiyama

Michael Hoch

Eric Hoffer

John Holemans

Morag Howard

Tianshen Hu

Dean Huang

Charles Hutchinson

Francesca Iacobellis

Isabella Iadevito

Massimo Imbriaco

Klaus Irion

Neeraj Jain

Judy James

Ehsan Jangholi

Eduardo Jayme

Sanjay Jeganathan

Devimeenal Jegannathan

Rajesh Jena

Jobin Jose

Nirmal Kakani

Chandan Kakkar

Thomas Kane

Karunakaravel Karuppasamy

Joachim Kettenbach

Sairah Khan

Rohit Khandelwal

Amithavikrama Kheda

Osama Kheiralla

Claudia Kirsch

Anwer Sadat Kithir Mohamed

Willemijn Klein

Michail Klontzas

Stephen Knight

Zehra Pinar Koc

Tuomas Koivumaki

Digna Kool

Wilhelm Kuker

Akira Kunimatsu

Emanuele La Corte

Hans-Ulrich Laasch

Susan Lalondrelle

Francesco Lassandro

Justin Lee

Steven Lev

Annemieke Littooij

Giuseppe Lo Re

Vibeke Loegager

Elaine Lui

Matthew Lukies

Eugen Lungu

Iain Lyburn

David Macdonald

Francesco Macrì

Grant Mair

Shashikant Mane

Lucia Manganaro

Narayana Manupratap

Magda Marcon

Maria Adele Marino

Benjamin Martin

Carine Martins Jarnalo

Rishi Mathew

Anthony Maxwell

Marius Mayerhofer

Michalis Mazonakis

Kieran McHugh

Hakim Megadmi

Shibani Mehra

Iosif Mendichovszky

Liesbeth Meylaerts

Dipayan Mitra

Hosahalli Mohan

Sally Morgan-Fletcher

Shinichiro Mori

Aun Muhammad

Nicola Mulholland

Jader Muller

Karl Murphy

Sujit Nair

Malavika Nathan

Selim Nural

Curtis Offiah

Akin Öğretici

Cem Onal

Davide Orlandi

Jose Otero

Kavitha Palled

Jyoti Parikh

David Parker

Tufail Patankar

Vishal Patel

Aruna Patil

Micheal Patlas

Marko Pecina

Deborah Pencharz

Robert Percarpio

Kostas Perisinakis

Martina Pezzullo

Dario Picone

Armando Pingitore

Antonio Pinto

Ahmet Veysel Polat

Gillian Potter

Satish Prasad

Mark Radon

Ashok Raghavan

Raili Raininko

KV Rajagopal

Hamid Rajebi

Victor Ramos Pacheco

Sameer Raniga

Anuradha Rao

Abdel Razek

Jonathan Richenberg

Rainer Rienmueller

Giles Roditi

Federica Romano

Luigia Romano

Martha Romero

Eugenio Rossi

Carmela Russo

Suzanne Ryan

Zia Saad

Anju Sahdev

Rakesh Sajjan

Osamu Sakai

David Sallomi

Saurabh Samdariya

Deepali Saxena

Ian Sayers

Rüdiger Schernthaner

Michele Scialpi

Debraj Sen

Pietro Sergio

Manjunath Shekar

Trishna Shimpi

Shivakumar Swamy Shivalingappa

Phillip Shorvon

Pelagie Sikiminywa-Kambale

Abhishek Solanki

Ali Solmaz

Vijay Somasundaram

Aleksandra Stankiewicz

Alessandro Stecco

David Stobo

Sam Stuart

Mukesh Surya

Turgut Tali

Henry Tam

Stefano Tartari

Shanigarn Thiravit

Richard Thomas

Adam Thomas

John Thompson

Mark Thornton

Damian Tolan

Christine Tolman

Anderanik Tomasian

Noriaki Tomura

Michele Tonerini

Sarah Townsend

Ken Tung

Yusuke Uchiyama

Calper Usluogullari

Rick van Rijn

Joost van Schuppen

V R Krishna Varanasi

Alejandro Vega Gutierrez

Antonello Vidiri

Sobhan Vinjamuri

Richard West

Elspeth Whitby

Thida Win

Stefan Wirth

Guangzhao Yang

Kent Yip

Akira Yogi

Dominic Yu

Qingqiang Zhu

